# Vitamin D and Metabolic Dysfunction-Associated Steatotic Liver Disease: Molecular Mechanisms and Clinical Implications—A Narrative Review

**DOI:** 10.3390/ijms27062532

**Published:** 2026-03-10

**Authors:** Héctor Fuentes-Barría, Raúl Aguilera-Eguía, Miguel Alarcón-Rivera, Lisse Angarita-Davila, Cherie Flores-Fernández

**Affiliations:** 1 Centro de Investigación en Medicina de Altura (CEIMA), Universidad Arturo Prat, Iquique 1110939, Chile; 2 Departamento de Salud Pública, Facultad de Medicina, Universidad Católica de la Santísima Concepción, Concepción 3349001, Chile; raguilerae@ucsc.cl; 3 Escuela de Ciencias del Deporte y Actividad Física, Facultad de Salud, Universidad Santo Tomas, Talca 3460000, Chile; mrivera3@santotomas.cl; 4 Escuela de Nutrición y Dietética, Facultad de Medicina, Universidad Andres Bello, Concepción 3349001, Chile; lisse.angarita@unab.cl; 5 Departamento de Gestión de la Información, Universidad Tecnológica Metropolitana, Santiago 7550000, Chile; cflores@utem.cl

**Keywords:** vitamin D, non-alcoholic fatty liver disease, fatty liver, molecular biology

## Abstract

Vitamin D has been extensively investigated for its role in Metabolic Dysfunction-Associated Steatotic Liver Disease (MASLD), a chronic condition characterized by hepatic steatosis, insulin resistance, inflammation, and metabolic dysregulation. This review examines the molecular mechanisms through which vitamin D influences liver metabolism, insulin signaling, lipid accumulation, and inflammatory pathways while evaluating its potential clinical applications in MASLD management. In its active form, 1,25-dihydroxyvitamin D3, vitamin D modulates hepatocyte function by reducing proinflammatory cytokines, enhancing insulin sensitivity, activating AMPK signaling, inhibiting mTOR pathways, and regulating lipid homeostasis. These effects contribute to decreased hepatic fat deposition and improved metabolic profiles, which are key in MASLD progression. Evidence also suggests that vitamin D supplementation may improve liver enzymes, insulin resistance, and lipid parameters in patients with MASLD, although responses vary depending on dosage, baseline vitamin D status, and patient characteristics. Despite promising findings, inconsistencies in study design, measurement methods, and population differences underscore the need for standardized approaches and personalized strategies. In conclusion, vitamin D demonstrates complementary therapeutic potential in MASLD, highlighting research gaps related to optimal dosing, duration, and long-term outcomes. Future studies should integrate mechanistic insights with clinical trials to optimize vitamin D’s role in improving liver and metabolic health.

## 1. Introduction

Vitamin D is increasingly recognized as a key regulator of human health due to its pleiotropic actions extending beyond classical bone metabolism [1]. Accumulating evidence suggests that vitamin D signaling plays a critical role in the pathophysiology of chronic metabolic diseases, including non-alcoholic fatty liver disease (NAFLD), which was recently redefined as metabolic dysfunction-associated steatotic liver disease (MASLD), a condition characterized by hepatic lipid accumulation, insulin resistance, inflammation, and progressive fibrogenesis [2,3].

The worldwide global prevalence of MASLD is estimated to be as high as 30% [4]. Notably, Global Burden of Disease (GBD) 2021 data indicate that among adolescents and young adults (15–39 years), approximately 423 million cases were reported in 2021, representing a 75.31% increase since 1990, with an age-standardized prevalence rate of 14,221.32 per 100,000 and a projected rise to 16,101 per 100,000 by 2050, highlighting the rapidly escalating global burden of this condition [5].

While vitamin D deficiency has been consistently associated with metabolic dysfunction, the molecular mechanisms linking vitamin D signaling to hepatic steatosis, inflammation, and fibrosis remain incompletely understood [6,7]. In this context, among the pleiotropic effects of vitamin D, its regulatory role in zinc homeostasis has gained increasing attention, as zinc functions as an essential cofactor for antioxidant defense systems, immune modulation, and the maintenance of hepatocellular integrity [8]. Disruption of zinc balance has been associated with heightened oxidative stress, activation of inflammatory responses, and the development of hepatotoxicity, factors that may accelerate disease progression in metabolic liver disorders [9].

MASLD represents the hepatic manifestation of metabolic syndrome and is closely associated with obesity, insulin resistance, and type 2 diabetes mellitus [10]. Its prevalence has increased dramatically worldwide, paralleling the rise in sedentary lifestyles and energy-dense dietary patterns [11]. Importantly, MASLD encompasses a disease spectrum ranging from simple steatosis to Metabolic Dysfunction-Associated Steatohepatitis (MASH), advanced fibrosis, cirrhosis, and hepatocellular carcinoma [12,13]. Despite its global burden, effective pharmacological therapies remain limited, highlighting the need to identify novel molecular targets and non-pharmacological strategies [14].

In this context, vitamin D has emerged as a potential metabolic modulator in MASLD [15]. Epidemiological studies have reported an inverse association between serum 25-hydroxyvitamin D levels and hepatic fat accumulation, liver enzyme elevation, and fibrosis severity [16,17]. Experimental evidence suggests that vitamin D, through activation of the vitamin D receptor (VDR), may influence key pathogenic processes in MASLD, including insulin sensitivity, lipid metabolism, inflammatory signaling, oxidative stress, and hepatic stellate cell activation [18]. However, clinical findings remain heterogeneous, raising questions regarding dose–response relationships, baseline vitamin D status, genetic variability, and disease stage [19].

At the molecular level, vitamin D signaling has been shown to attenuate pro-inflammatory pathways such as NF-κB, reduce oxidative stress, modulate autophagy, and regulate adipogenesis and lipid flux between adipose tissue and the liver [20,21,22]. These effects position vitamin D as a potential regulator of the liver–adipose tissue axis, a central component in MASLD progression [23,24]. Nevertheless, the translation of these molecular effects into clinically meaningful outcomes remains controversial [25].

Therefore, this narrative review aims to synthesize current evidence on the molecular mechanisms through which vitamin D signaling influences hepatic lipid metabolism, inflammation, insulin resistance, and fibrogenic pathways in MASLD, while critically evaluating its therapeutic potential and limitations in clinical practice.

## 2. Pathophysiology of MASLD: Molecular and Metabolic Basis

MASLD, is a spectrum of chronic liver disorders characterized by excessive hepatic lipid accumulation in the absence of significant alcohol consumption or other secondary causes of steatosis [26]. MASLD is currently the most prevalent liver disease worldwide and represents the hepatic manifestation of metabolic syndrome, closely associated with obesity, insulin resistance, dyslipidemia, and type 2 diabetes mellitus [10]. Clinically, MASLD encompasses a wide pathological continuum ranging from simple steatosis to MASH, progressive fibrosis, cirrhosis, and hepatocellular carcinoma, with fibrosis stage being the strongest predictor of liver-related morbidity and mortality [12,13,27].

Epidemiological studies reveal important demographic differences in MASLD/NAFLD. Sex differences are evident, with higher prevalence observed in men compared to women in most age groups; however, this gap narrows after menopause, consistent with a protective effect of estrogens in pre-menopausal women [28]. Older age is also associated with increased prevalence and severity of hepatic steatosis and fibrosis, making age a strong determinant of disease burden [29]. Additionally, race and ethnicity influence MASLD risk and severity: Hispanic populations consistently show the highest prevalence and advanced steatosis, while individuals of African descent tend to have lower rates of MASLD despite high metabolic risk burden, likely reflecting genetic and adiposity distribution differences [30]. These patterns underscore how biological sex, aging, and ancestry contribute to MASLD heterogeneity and should be considered in risk stratification and management [28].

In its early stages, MASLD is frequently asymptomatic, contributing to underdiagnosis and delayed clinical intervention [31]. When present, clinical manifestations are often nonspecific and may include fatigue, right upper quadrant discomfort, and mild hepatomegaly [32]. As the disease progresses, chronic hepatic injury can lead to portal hypertension, hepatic insufficiency, and increased cardiovascular risk, which represents the leading cause of mortality in this population [33]. Importantly, MASLD is increasingly recognized as a multisystem disease, exerting deleterious effects beyond the liver, including increased risk of chronic kidney disease, cardiovascular events, and extrahepatic malignancies [33,34].

From a pathophysiological standpoint, hepatic steatosis arises from an imbalance between lipid acquisition and disposal within hepatocytes [35]. This process is primarily driven by insulin resistance, which promotes increased flux of free fatty acids from adipose tissue to the liver, enhanced de novo lipogenesis, impaired mitochondrial β-oxidation, and increased VLDL secretion as a compensatory mechanism to export excess triglycerides; however, this secretory capacity is insufficient to counterbalance the marked lipid oversupply, ultimately resulting in intrahepatic lipid accumulation [36,37].

Importantly, the relationship between hepatic lipid accumulation and insulin resistance is bidirectional. While systemic insulin resistance promotes increased lipid delivery to the liver, excessive intrahepatic lipid deposition—particularly diacylglycerols and ceramides—can directly impair insulin signaling within hepatocytes. These lipotoxic intermediates activate protein kinase C isoforms and stress-related kinases, leading to inhibition of insulin receptor substrate phosphorylation and disruption of downstream PI3K/Akt signaling. Thus, in early stages of MASLD, lipid overload itself may be a primary driver of hepatic insulin resistance, establishing a self-perpetuating cycle that exacerbates metabolic dysfunction and disease progression.

Chronic exposure to excess lipids induces lipotoxicity, mitochondrial dysfunction, and oxidative stress, triggering hepatocellular injury and activation of inflammatory pathways [38]. These alterations contribute to a silent but progressive disease course, often remaining clinically undetected until advanced stages are reached pathways [31].

Environmental and lifestyle factors play a central role in the development and progression of MASLD [39]. An obesogenic environment characterized by excessive caloric intake, sedentary behavior, and visceral adipose tissue accumulation exacerbates hepatic insulin resistance and promotes chronic low-grade inflammation [40]. Dysfunctional adipose tissue contributes to increased release of free fatty acids and pro-inflammatory adipokines, such as leptin, while reducing protective factors such as adiponectin [41]. This altered adipokine profile amplifies hepatic inflammation and fibrogenesis, thereby accelerating MASLD progression and increasing the risk of long-term complications [42,43].

At the molecular level, MASLD is associated with profound alterations in intracellular signaling pathways regulating metabolism, inflammation, and cell survival [42]. Dysregulation of insulin signaling pathways, including PI3K/Akt and AMPK, promotes lipid accumulation and impairs glucose and fatty acid homeostasis [44]. Concurrently, activation of stress-sensitive pathways, such as NF-κB and JNK, together with excessive production of reactive oxygen species, perpetuates inflammatory signaling and hepatocellular damage [45]. These molecular disturbances also stimulate hepatic stellate cell activation through profibrogenic mediators such as transforming growth factor-β, leading to extracellular matrix deposition and fibrosis [46].

The prevention and management of MASLD have traditionally focused on lifestyle modification as the cornerstone of therapy [11,39]. Weight reduction through dietary intervention and regular physical activity remains the most effective strategy for reducing hepatic steatosis and improving metabolic parameters [28]. Medical nutrition therapy aims to achieve sustained caloric deficit while optimizing macronutrient composition, typically emphasizing reduced intake of refined carbohydrates and saturated fats, adequate protein consumption, and increased dietary fiber [47]. When combined with physical activity, these interventions improve insulin sensitivity, reduce hepatic fat content, and may slow disease progression, although their long-term adherence remains challenging [48].

Table 1 provides a comprehensive overview of MASLD, summarizing its clinical spectrum, underlying metabolic and molecular mechanisms, and associated long-term complications. The table highlights key features of disease progression, from simple steatosis to advanced fibrosis and cirrhosis, as well as the systemic consequences linked to chronic metabolic dysfunction.

**Figure 1:**
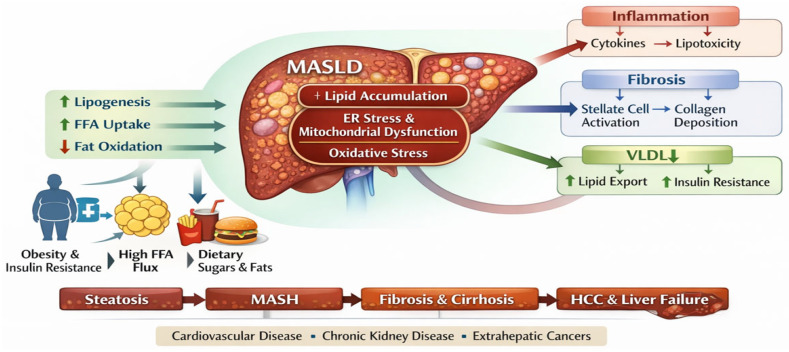
Pathophysiology of MASLF. Source: own elaboration.

Figure 1 provides an integrated overview of the pathophysiological mechanisms underlying MASLD, highlighting the imbalance between hepatic lipid acquisition and disposal as the central event. The figure illustrates the major sources of free fatty acids, including increased adipose tissue lipolysis in the context of insulin resistance and enhanced de novo lipogenesis, together with reduced mitochondrial β-oxidation and impaired triglyceride export as very-low-density lipoproteins (VLDLs). This lipid overload promotes lipotoxicity, endoplasmic reticulum stress, mitochondrial dysfunction, and excessive production of reactive oxygen species, which in turn activate inflammatory and profibrogenic pathways driving progression from simple steatosis to MASH, fibrosis, cirrhosis, and hepatocellular carcinoma. Additionally, the figure emphasizes the modulatory role of environmental and metabolic factors—such as obesity, a sedentary lifestyle, and adipose tissue dysfunction—in amplifying hepatic injury and linking MASLD to systemic complications, particularly cardiovascular and renal diseases.

## 3. Vitamin D

### 3.1. Molecular and Metabolic Basis

Vitamin D is a fat-soluble secosteroid hormone obtained through cutaneous synthesis following ultraviolet B (UVB) radiation exposure or, to a lesser extent, through dietary intake [52]. It is estimated that approximately 80–90% of circulating vitamin D derives from endogenous skin production, whereas only about 10–20% is obtained from dietary sources under usual conditions [53]. Endogenous production in the skin accounts for the majority of circulating vitamin D, where 7-dehydrocholesterol is converted into previtamin D_3_ and subsequently isomerized into cholecalciferol [54,55]. Dietary sources include vitamin D_3_ (cholecalciferol) of animal origin and vitamin D_2_ (ergocalciferol) derived from plant sources and fortified foods [49]. Regardless of its origin, vitamin D is biologically inactive and requires sequential hydroxylation steps to exert its physiological effects [55].

The first hydroxylation step occurs primarily in the liver, where vitamin D is converted into 25-hydroxyvitamin D [25(OH)D], the major circulating form and the most reliable biomarker of vitamin D status [19]. This process is mediated mainly by hepatic cytochrome P450 enzymes, including CYP2R1 and CYP27A1 [56,57,58]. Given the central role of the liver in vitamin D metabolism, hepatic dysfunction may significantly impair vitamin D activation and bioavailability [59]. In chronic liver diseases such as MASLD, alterations in hepatocellular function, lipid accumulation, and inflammation may disrupt normal vitamin D metabolism, contributing to the high prevalence of vitamin D deficiency observed in these patients [59,60].

The second hydroxylation step takes place predominantly in the kidneys, where 25(OH)D is converted into the biologically active form 1,25-dihydroxyvitamin D [1,25(OH)_2_D] by the enzyme 1α-hydroxylase (CYP27B1) [56,57]. Although the kidney is the principal site of this conversion, extra-renal expression of CYP27B1 has been identified in multiple tissues, including immune cells and hepatic cells, suggesting that vitamin D may exert local autocrine and paracrine effects [56,57]. This tissue-specific activation highlights the complexity of vitamin D metabolism and supports its role as a pleiotropic regulator of metabolic and immune functions [61].

Circulating vitamin D metabolites are transported while bound to vitamin D-binding protein (DBP), which regulates their bioavailability and tissue distribution [62]. Alterations in DBP levels or binding affinity, as observed in inflammatory and metabolic conditions, may further influence vitamin D status independently of total serum 25(OH)D concentrations [62,63]. In the context of obesity and MASLD, vitamin D sequestration within adipose tissue has been proposed as an additional mechanism contributing to reduced circulating levels, thereby limiting its biological availability despite adequate intake or sun exposure [63,64].

The biological actions of vitamin D are primarily mediated through binding to the vitamin D receptor (VDR), a member of the nuclear receptor superfamily expressed in a wide range of tissues [65]. Upon activation by 1,25(OH)_2_D, VDR forms a heterodimer with the retinoid X receptor and binds to vitamin D response elements within target gene promoters, regulating transcription of genes involved in calcium homeostasis, metabolism, immune modulation, and cell differentiation [66]. In addition to these classical genomic actions, vitamin D can also trigger rapid non-genomic signaling pathways involving kinases such as MAPK, PI3K/Akt, and AMPK, which are particularly relevant in metabolic tissues [67].

Importantly, VDR expression has been demonstrated in multiple hepatic cell types, including hepatocytes, Kupffer cells, hepatic stellate cells, and cholangiocytes [68]. This widespread hepatic expression supports a direct role for vitamin D signaling in liver physiology and pathophysiology [18,23,69]. In hepatocytes, VDR activation has been implicated in the regulation of lipid and glucose metabolism, whereas in immune and stellate cells it modulates inflammatory and fibrogenic responses [70]. These cell-specific actions position vitamin D as an integrative regulator within the hepatic microenvironment, capable of influencing multiple pathogenic pathways involved in MASLD progression [71].

Vitamin D homeostasis is tightly regulated through feedback mechanisms involving parathyroid hormone, fibroblast growth factor 23, and the catabolic enzyme 24-hydroxylase (CYP24A1), which inactivates both 25(OH)D and 1,25(OH)_2_D [72,73]. Dysregulation of these pathways may further contribute to altered vitamin D signaling in metabolic diseases [56]. In MASLD, chronic inflammation and oxidative stress may affect the expression and activity of vitamin D-metabolizing enzymes and VDR itself, potentially attenuating downstream signaling despite adequate circulating levels [20,74].

**Figure 2:**
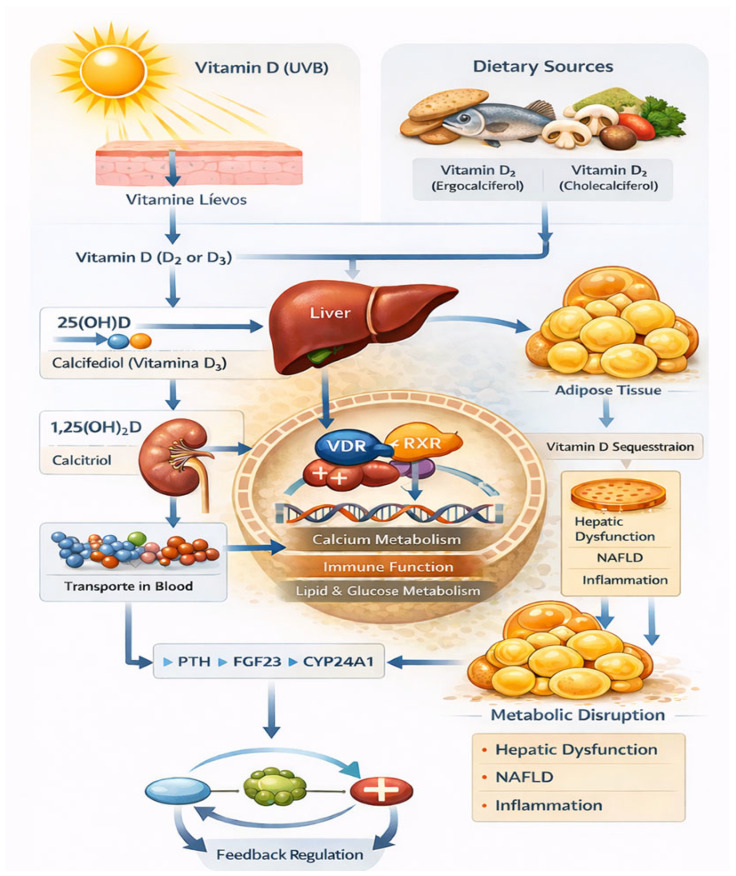
Vitamin D metabolism. Source: own elaboration.

Overall, vitamin D metabolism represents a complex, multistep process that is highly dependent on hepatic function and metabolic health. Disruptions at any level of this pathway—from cutaneous synthesis and hepatic hydroxylation to receptor activation and intracellular signaling—may compromise vitamin D-mediated biological effects. Understanding how MASLD-associated metabolic alterations interfere with vitamin D metabolism and signaling is essential for clarifying its role in disease pathogenesis and for optimizing therapeutic strategies targeting the vitamin D–VDR axis (Figure 2).

### 3.2. Vitamin D, Insulin Resistance, and Hepatic Glucose–Lipid Crosstalk

Vitamin D exerts a wide range of pleiotropic effects that directly influence metabolic pathways involved in insulin resistance and glucose–lipid homeostasis, processes that are central to the pathogenesis of MASLD [75]. MASLD is strongly associated with systemic and hepatic insulin resistance, a condition that promotes excessive hepatic glucose production and lipid accumulation [76].

The lipid aggregates that accumulate within hepatocytes consist predominantly of triglycerides stored in cytoplasmic lipid droplets, derived from increased adipose tissue lipolysis, enhanced de novo lipogenesis, and dietary fatty acid influx [77]. These droplets may also contain cholesterol esters, free cholesterol, diacylglycerols, and ceramides, lipid species that are closely linked to lipotoxicity, mitochondrial dysfunction, and pro-inflammatory signaling [78]. In this context, several studies have demonstrated an inverse association between circulating vitamin D levels and insulin resistance indices, such as the Homeostatic Model Assessment of Insulin Resistance (HOMA-IR), particularly in individuals with increased body mass index and hepatic steatosis [79,80]. These findings suggest that vitamin D deficiency may contribute to metabolic dysregulation at the hepatic level, exacerbating MASLD progression [81]. From a therapeutic perspective, although no pharmacological agent is universally approved specifically for MASLD, insulin sensitizers, GLP-1 receptor agonists, SGLT2 inhibitors, and antioxidant therapy with vitamin E have shown efficacy in reducing hepatic steatosis and improving metabolic parameters, thereby mitigating the harmful effects of lipid accumulation [82,83,84].

At the molecular level, vitamin D influences insulin sensitivity and glucose metabolism through mechanisms that extend beyond pancreatic β-cell function and involve direct actions within hepatic tissue [85]. Activation of the VDR regulates the transcription of genes involved in insulin signaling pathways, including those associated with insulin receptor expression, intracellular signal transduction, and glucose transporter activity [75,86].

Specifically, experimental studies have shown that vitamin D–VDR signaling upregulates insulin receptor substrate-1 (IRS-1) expression and enhances phosphorylation of the PI3K/Akt pathway, thereby improving downstream metabolic signaling in hepatocytes [87]. VDR activation has also been associated with increased AMPK activity and suppression of inflammatory mediators such as TNF-α and NF-κB, which are known to impair insulin signaling [88]. Through modulation of these key molecular targets, vitamin D contributing to improved insulin responsiveness and suppression of inappropriate hepatic gluconeogenesis, a hallmark of insulin-resistant states [89].

Hepatic insulin resistance disrupts the tightly regulated balance between glucose and lipid metabolism, promoting increased de novo lipogenesis while failing to suppress glucose output [90]. Vitamin D has been shown to influence this metabolic crosstalk by modulating pathways that integrate insulin signaling with lipid metabolism [91]. VDR activation enhances the expression and activity of proteins involved in insulin signaling, such as insulin receptor substrates and downstream kinases, thereby facilitating insulin-mediated metabolic control in hepatocytes [92]. Through these mechanisms, vitamin D may contribute to reduced hepatic lipid accumulation and improved metabolic flexibility [93].

However, interindividual variability in response to vitamin D supplementation has been reported, partially attributed to genetic polymorphisms affecting vitamin D metabolism and signaling [94]. Single nucleotide polymorphisms (SNPs) in genes such as *CYP27B1*, *CYP2R1*, and VDR have been associated with differences in circulating 25-hydroxyvitamin D levels and downstream metabolic effects [48,49]. In MASLD, these genetic variations may influence the extent to which vitamin D can modulate hepatic insulin sensitivity and lipid metabolism [95]. Although polymorphisms such as *rs10877012* in *CYP27B1* have been linked to altered vitamin D status, genetic stratification is not yet routinely implemented in clinical practice due to cost, complexity, and limited evidence supporting personalized supplementation strategies [56,57].

Vitamin D deficiency is consistently associated with impaired glucose tolerance and exacerbation of insulin resistance at the hepatic level [96]. In MASLD, reduced vitamin D availability contributes to impaired insulin-mediated suppression of hepatic glucose production, further aggravating hyperglycemia and metabolic stress [96,97]. This condition is compounded by insulin resistance in peripheral tissues, which increases circulating free fatty acid flux to the liver, reinforcing hepatic lipid overload and metabolic dysfunction [97,98]. Regarding insulin receptor expression, vitamin D has been shown to upregulate insulin receptor expression and enhance insulin receptor sensitivity in metabolic tissues, including the liver [97]. Acting as an epigenetic regulator, vitamin D promotes transcriptional activation of insulin receptor-related genes, leading to improved downstream signaling efficiency [99]. Experimental evidence suggests that vitamin D increases the activity of insulin receptor substrates and enhances insulin responsiveness, thereby contributing to improved hepatic glucose and lipid homeostasis [75,97,99].

Chronic inflammation is a critical driver of insulin resistance in MASLD, and vitamin D deficiency has been associated with increased expression of proinflammatory cytokines that interfere with insulin signaling [59,63]. Obesity, a major risk factor for MASLD, is frequently accompanied by hypovitaminosis D due to reduced sun exposure, low dietary intake, and sequestration of vitamin D in adipose tissue [100]. In this inflammatory milieu, dysregulated adipokine secretion, including elevated leptin levels, further contributes to hepatic insulin resistance [101]. Emerging evidence suggests that vitamin D supplementation may reduce circulating leptin levels and improve insulin sensitivity, potentially through central and peripheral mechanisms involving VDR activation [102].

Vitamin D also exerts potent anti-inflammatory effects by modulating cytokine production and immune cell activity. It suppresses proinflammatory mediators such as interleukin-6 and tumor necrosis factor-α, which are implicated in hepatic insulin resistance and MASLD progression [103]. Additionally, vitamin D influences adipokine balance by increasing adiponectin levels, a hormone with anti-inflammatory and insulin-sensitizing properties [104]. Higher adiponectin concentrations are associated with improved hepatic insulin sensitivity and reduced steatosis, reinforcing the link between vitamin D status and metabolic liver health [105,106].

At the intracellular level, vitamin D interacts with key metabolic regulators, including AMP-activated protein kinase (AMPK) and the mammalian target of rapamycin (mTOR), which are central to hepatic energy sensing and nutrient metabolism [107,108]. AMPK activation promotes fatty acid oxidation and inhibits lipogenesis, thereby alleviating hepatic lipid accumulation and improving insulin sensitivity [109,110]. Vitamin D–VDR signaling has been shown to activate AMPK, particularly under conditions of metabolic stress, leading to enhanced lipid oxidation and reduced insulin resistance in hepatic cells [109,110].

Conversely, dysregulation of mTOR signaling contributes to hepatic insulin resistance and steatosis by promoting anabolic processes such as lipogenesis [111,112]. Vitamin D has been reported to modulate mTORC1 activity, preventing its excessive activation and thereby limiting lipid accumulation and inflammatory signaling in the liver [112]. By restoring the balance between AMPK and mTOR pathways, vitamin D may exert a protective role against metabolic dysregulation in MASLD [113,114].

Vitamin D also influences mTORC2 and the PI3K/Akt pathway, both of which are critical for insulin signaling and glucose metabolism. Activation of PI3K/Akt improves insulin-mediated suppression of gluconeogenesis and enhances metabolic control in hepatocytes [115,116]. Through VDR-dependent mechanisms, vitamin D promotes Akt phosphorylation and supports insulin sensitivity, contributing to improved hepatic glucose–lipid crosstalk. Genetic variability within these pathways may further modulate individual responses to vitamin D signaling, suggesting potential avenues for personalized interventions [116,117].

Beyond classical signaling pathways, vitamin D acts as an epigenetic regulator of glucose and lipid metabolism through modulation of microRNA (miRNA) expression. miRNAs play a crucial role in regulating inflammation, insulin signaling, and lipid metabolism, all of which are central to MASLD pathophysiology [15]. Vitamin D has been shown to regulate miRNAs such as miR-21 and miR-155, both of which are implicated in inflammatory signaling and insulin resistance. Suppression of miR-21 by vitamin D reduces nuclear factor κB activation, thereby attenuating hepatic inflammation, while downregulation of miR-155 favors an anti-inflammatory macrophage phenotype within the hepatic microenvironment [118,119].

**Figure 3:**
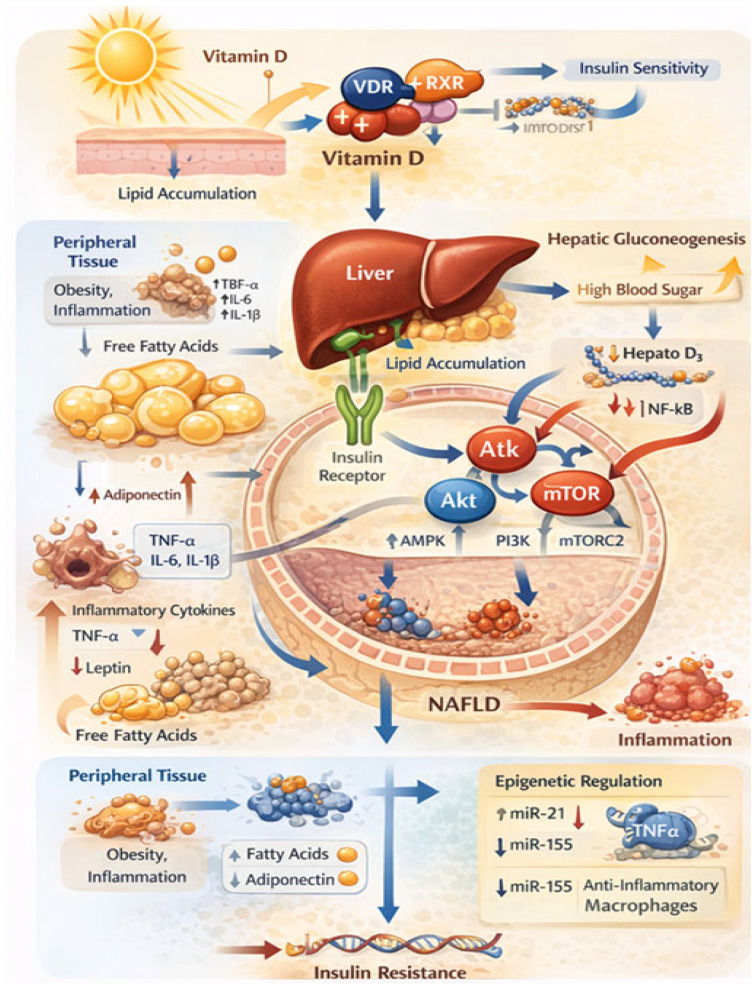
Illustrates the role of vitamin D in hepatic glucose–lipid crosstalk, highlighting its interaction with insulin signaling pathways, AMPK and mTOR regulation, inflammatory mediators, and epigenetic mechanisms involved in MASLD progression. Source: own elaboration.

These epigenetic mechanisms highlight the capacity of vitamin D to modulate hepatic insulin sensitivity and metabolic homeostasis at multiple regulatory levels. In addition, the interaction between adipocytes, inflammatory mediators, and hepatic signaling pathways contributes to lipid accumulation, altered insulin signaling, and the progression toward metabolic dysfunction. As illustrated in Figure 3, vitamin D signaling through the VDR/RXR complex influences insulin sensitivity, hepatic gluconeogenesis, and inflammatory pathways, while adipocyte-derived factors such as free fatty acids and cytokines further impact liver metabolism and insulin resistance. Nevertheless, further studies are required to elucidate how vitamin D supplementation influences miRNA networks in human MASLD and whether these effects translate into sustained clinical benefits.

### 3.3. Vitamin D, Adipogenesis, and the Liver–Adipose Axis in MASLD

Vitamin D plays a critical role in the regulation of adipose tissue biology, fat storage, and systemic energy homeostasis, thereby exerting indirect but profound effects on hepatic lipid metabolism [75,97,99]. Through activation of the vitamin D receptor (VDR), vitamin D modulates peroxisome proliferator-activated receptor gamma (PPARγ), a master regulator of adipocyte differentiation, lipid handling, and adipose tissue expandability [120,121,122]. Proper PPARγ activation promotes the differentiation of metabolically competent adipocytes and facilitates lipid sequestration within adipose tissue, limiting ectopic lipid deposition in the liver and other insulin-sensitive organs. This redistribution of lipids toward peripheral adipose depots represents a key protective mechanism against hepatic steatosis and lipotoxicity [123,124,125,126].

Adipogenesis is the tightly regulated differentiation process through which mesenchymal stem cells (MSCs) commit to the adipocyte lineage, ultimately giving rise to mature lipid-storing cells [127,128]. This process is essential for maintaining metabolic flexibility, particularly under conditions of caloric excess. During adipogenesis, MSCs undergo proliferation, clonal expansion, and differentiation into preadipocytes, which retain high plasticity before maturing into fully differentiated adipocytes [117,128]. Mature adipocytes are characterized by a unilocular lipid droplet occupying most of the cytoplasm, with the nucleus displaced toward the periphery and a relatively reduced mitochondrial and Golgi apparatus content, reflecting their specialization in lipid storage [129].

At the molecular level, adipogenesis proceeds through a well-defined transcriptional cascade. Early adipogenic commitment is initiated by the activation of transcription factors such as activator protein-1 (AP-1), CCAAT/enhancer-binding protein β (C/EBPβ), and C/EBPδ. These factors induce the expression of late adipogenic regulators, including PPARγ and C/EBPα, which cooperatively drive terminal adipocyte differentiation and establish the metabolic phenotype of mature adipocytes. Vitamin D signaling intersects with this transcriptional network by modulating PPARγ activity, thereby influencing both the rate and quality of adipocyte differentiation [130].

From a metabolic perspective, adipogenesis serves a protective function by enabling adipose tissue expansion through hyperplasia rather than hypertrophy. The formation of new, smaller adipocytes enhances lipid-buffering capacity and reduces the reliance on hypertrophic adipocytes, which are metabolically dysfunctional and exhibit a pro-inflammatory, hypoxic profile [131,132,133,134,135].

In obesity and vitamin D deficiency, impaired adipogenesis favors adipocyte hypertrophy, increased lipolysis, and excessive release of free fatty acids (FFAs) into circulation [136]. These FFAs are delivered to the liver, where they promote triglyceride accumulation, hepatic insulin resistance, and oxidative stress—key drivers of MASLD progression [137,138].

Vitamin D deficiency exacerbates adipose tissue dysfunction by impairing adipocyte differentiation, promoting chronic low-grade inflammation, and disrupting adipokine secretion [139,140]. Dysfunctional adipocytes secrete increased levels of pro-inflammatory cytokines while exhibiting reduced adiponectin production, thereby amplifying systemic insulin resistance and hepatic lipid accumulation [141]. In contrast, adequate vitamin D status supports healthy adipose tissue expansion, attenuates inflammation, and reduces FFA flux to the liver, highlighting the importance of the adipose–liver axis in MASLD pathophysiology [136].

Adipose tissue heterogeneity further refines this interaction [141]. Three main adipocyte phenotypes have been described: white adipocytes, specialized in triglyceride storage; brown adipocytes, which dissipate energy through thermogenesis; and beige adipocytes, an inducible subtype with intermediate characteristics [139,140]. Beige adipocytes possess enhanced mitochondrial content and thermogenic capacity, contributing to increased energy expenditure and reduced lipid spillover [132]. Emerging evidence suggests that vitamin D may favor the differentiation or maintenance of beige adipocytes under specific metabolic conditions, thereby decreasing circulating FFAs and lowering hepatic lipid burden [136,137,138].

In addition to its effects on adipocyte differentiation, vitamin D regulates autophagy within adipose tissue, a process essential for adipocyte remodeling, lipid turnover, and cellular homeostasis [132]. Through VDR-mediated gene regulation, vitamin D influences autophagic flux, preventing excessive or dysfunctional adipogenesis that can lead to inflamed and fibrotic adipose tissue [3,17,71]. By maintaining balanced adipose tissue remodeling, vitamin D contributes to reduced systemic inflammation and improved metabolic signaling toward the liver [75].

Clinical evidence supports these mechanistic observations, with meta-analyses reporting modest but significant effects of vitamin D supplementation on body weight, waist circumference, and fat distribution, particularly in individuals with baseline vitamin D deficiency [142,143,144]. Although changes in anthropometric measures are relatively small, they reflect meaningful improvements in adipose tissue function and lipid partitioning, which may translate into reduced hepatic fat accumulation and improved metabolic outcomes over time [145].

Collectively, vitamin D emerges as a key regulator of adipogenesis and adipose tissue function, with downstream effects on hepatic lipid metabolism and MASLD progression [145]. By promoting healthy adipocyte differentiation, supporting PPARγ-mediated lipid redistribution, and potentially enhancing beige adipocyte activity, vitamin D helps limit FFA overflow to the liver and attenuate hepatic steatosis [146]. These findings underscore the importance of the liver–adipose axis as a therapeutic target and position vitamin D as a systemic modulator of metabolic health in MASLD [145].

**Figure 4:**
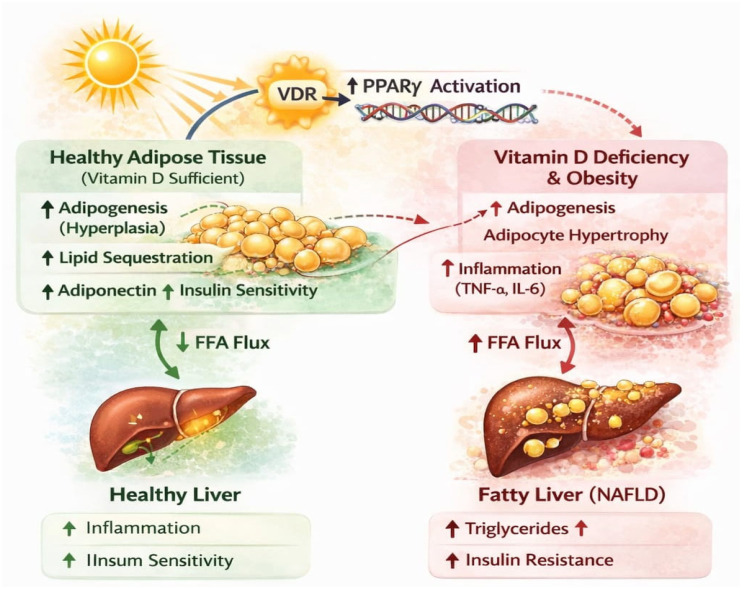
Illustrates the role Vitamin D, Adipogenesis, and the Liver–Adipose Axis in MASLD. Source: own elaboration.

Together, Figure 4 offers an integrated framework for understanding the molecular mechanisms underlying adipocyte differentiation, including the transcriptional programs that drive the formation of white, brown, and beige adipocytes. These processes critically influence lipid storage capacity, thermogenic activity, and systemic energy expenditure. Importantly, the balance between adipocyte phenotypes determines free fatty acid flux toward the liver, thereby modulating hepatic lipid accumulation, insulin sensitivity, and the progression of MASLD. As such, these visual and tabulated tools are essential for contextualizing how adipose tissue biology intersects with hepatic metabolism and overall metabolic health.

Complementarily, Table 2 provides a detailed overview of the key genetic and transcriptional regulators involved in adipogenesis and adipose tissue remodeling, highlighting their specific functions and metabolic implications within the liver–adipose axis. This table summarizes the roles of pivotal factors such as PPARγ, C/EBPα, C/EBPβ, PRDM16, and UCP1, among others, and illustrates how their coordinated activation governs adipocyte differentiation and phenotypic specification.

### 3.4. Vitamin D Status and Clinical Thresholds in Metabolic Liver Disease

Serum vitamin D status is commonly assessed by measuring circulating concentrations of 25-hydroxyvitamin D [25(OH)D], reported in either ng/mL or nmol/L. Despite its widespread use, there is no universally accepted consensus regarding the optimal cutoff points defining vitamin D deficiency, insufficiency, and sufficiency, particularly in the context of metabolic disorders [19]. According to the Institute of Medicine (IOM), vitamin D status is categorized as severe deficiency (10–12 ng/mL or 25–30 nmol/L), insufficiency (<20 ng/mL or <50 nmol/L), and adequacy (>20 ng/mL or >50 nmol/L) [2]. These thresholds, while primarily established based on bone health outcomes, are frequently applied in studies evaluating metabolic diseases, including MASLD [154].

The lack of standardized criteria for defining vitamin D sufficiency has generated variability across clinical guidelines and research studies. Organizations such as the International Osteoporosis Foundation and the American Geriatrics Society propose alternative thresholds, reflecting differences in the interpretation of available evidence [19]. These discrepancies arise from heterogeneity in analytical methodologies for 25(OH)D measurement, differences in study populations, genetic background, environmental exposure, and the clinical endpoints considered [19]. Importantly, bone-related outcomes may not adequately capture the vitamin D requirements necessary to modulate metabolic, inflammatory, and hepatic pathways involved in MASLD progression [69].

Emerging evidence suggests that individuals with metabolic dysfunction, obesity, insulin resistance, or chronic liver disease may require higher circulating levels of vitamin D to achieve optimal extra-skeletal effects [140,143,144,145,146]. In patients with MASLD, lower serum 25(OH)D concentrations have been consistently associated with increased hepatic fat accumulation, insulin resistance, elevated aminotransferases, and greater risk of progression toward MASH [136,145]. These observations support the notion that vitamin D thresholds defined for bone health may underestimate the levels required to exert protective effects on hepatic metabolism and inflammation [136,145].

Vitamin D deficiency represents a global public health concern, with particularly high prevalence among populations living at higher latitudes, where limited ultraviolet B (UVB) exposure reduces cutaneous vitamin D synthesis [2,155,156]. Additional risk groups include individuals with increased skin pigmentation, as higher melanin content reduces UVB-mediated vitamin D production, as well as individuals with obesity, in whom vitamin D is sequestered in adipose tissue, lowering its bioavailability [157,158]. These factors are highly relevant to MASLD, which frequently coexists with obesity and insulin resistance, further exacerbating hypovitaminosis D in this population [136,145].

Furthermore, lifestyle factors such as sedentary behavior, reduced outdoor activity, and dietary patterns low in vitamin D-rich foods contribute to suboptimal vitamin D status, particularly in individuals with metabolic syndrome [11,41,75]. Given the bidirectional relationship between vitamin D deficiency and metabolic dysfunction, inadequate vitamin D levels may not only be a consequence of MASLD but also a contributing factor to its development and progression [145].

Table 3 summarizes commonly used serum 25(OH)D cutoff values and their clinical interpretation, providing a standardized framework for categorizing vitamin D status in both general and metabolically compromised populations. This table facilitates the comparison of findings across studies and underscores the need for disease-specific thresholds when evaluating the role of vitamin D in MASLD and related metabolic disorders.

### 3.5. Vitamin D, AMPK–mTOR Signaling, and Obesity-Driven MASLD

Building upon the insulin-related mechanisms discussed in the previous sections, vitamin D-mediated regulation of energy-sensing pathways emerges as a critical link between obesity and MASLD progression [145]. In particular, the AMPK–mTOR axis integrates nutrient availability, adiposity, and inflammatory cues, thereby modulating hepatic lipid metabolism, autophagy, and insulin sensitivity in obesity-driven MASLD [147,149].

The interaction between vitamin D, AMPK, and the mammalian target of rapamycin (mTOR) constitutes a central regulatory axis linking energy sensing, adiposity, and hepatic lipid metabolism. This signaling network plays a pivotal role in the development of obesity-related metabolic dysfunction and the progression of MASLD [145,147,149]. Vitamin D, through VDR-mediated mechanisms, modulates both AMPK and mTOR pathways, thereby influencing lipid handling, insulin sensitivity, and inflammatory tone in adipose tissue and liver [65,66,109].

Vitamin D deficiency has been consistently associated with increased visceral adiposity, a major driver of metabolic dysfunction and hepatic steatosis [7,22]. Excess accumulation of abdominal fat promotes elevated free fatty acid flux to the liver, exacerbating hepatic triglyceride deposition, insulin resistance, and oxidative stress [36]. This process is further amplified by the chronic low-grade inflammatory state characteristic of vitamin D deficiency, which contributes to systemic metabolic dysregulation and MASLD progression [16,100,121,142,145].

At the molecular level, insufficient vitamin D impairs AMPK activation, a key cellular energy sensor that promotes fatty acid oxidation, inhibits lipogenesis, and enhances insulin sensitivity [159]. Through activation of AMPK, vitamin D supports metabolic flexibility in both adipose tissue and hepatocytes, facilitating lipid utilization and limiting ectopic fat accumulation [159]. Reduced AMPK activity in vitamin D-deficient states favors lipid storage, mitochondrial dysfunction, and increased susceptibility to insulin resistance, all of which are hallmarks of MASLD [145,159].

Concurrently, vitamin D influences the mTOR signaling pathway, a central regulator of cellular growth, nutrient sensing, and lipid synthesis [111]. Excessive mTOR activation, particularly mTORC1 has been implicated in adipocyte hypertrophy, increased lipogenesis, and impaired insulin signaling [112,149]. Vitamin D has been shown to suppress aberrant mTOR activity, thereby attenuating anabolic lipid accumulation and improving metabolic homeostasis [111]. By modulating the AMPK–mTOR balance, vitamin D contributes to reduced adiposity, improved adipose tissue function, and lower hepatic lipid burden [111,159].

Inflammation represents a critical link between obesity, vitamin D deficiency, and MASLD [59]. Vitamin D exerts anti-inflammatory effects within adipose tissue by modulating mTOR-dependent inflammatory pathways, reducing the expression of pro-inflammatory cytokines and promoting a healthier adipokine profile [111]. This anti-inflammatory action mitigates adipose tissue dysfunction, decreases systemic insulin resistance, and indirectly protects the liver from inflammation-driven progression toward MASH [160].

While vitamin D supplementation has demonstrated metabolic benefits, it is essential to consider safety and dosing [161]. Excessive vitamin D intake, although uncommon, can lead to hypervitaminosis D and hypercalcemia, primarily due to excessive supplementation [162,163]. Elevated vitamin D levels may overstimulate calcium homeostasis and parathyroid hormone activity, potentially resulting in cardiac arrhythmias, neurological disturbances, and renal complications [163]. Therefore, maintaining optimal—not excessive—vitamin D levels is critical to harness metabolic benefits while minimizing adverse effects [164].

Preventive and therapeutic strategies to address vitamin D deficiency in metabolic liver disease require a multifaceted approach [164]. Supplementation remains the most effective intervention for individuals at high risk, including those with obesity, limited sun exposure, or established MASLD [59]. Dietary strategies, such as increased consumption of vitamin D-rich foods (e.g., fatty fish, eggs, liver, and fortified dairy products), contribute to improving baseline vitamin D status and may support long-term metabolic health [25].

Controlled sun exposure represents an additional, physiologically relevant source of vitamin D synthesis; however, its effectiveness is influenced by latitude, season, skin pigmentation, and lifestyle factors [156,157,158]. Public health interventions, including food fortification policies, have proven effective in reducing vitamin D deficiency at the population level and may indirectly contribute to lowering the burden of metabolic diseases [161].

Collectively, these strategies highlight the importance of maintaining adequate vitamin D status as part of an integrated approach to preventing obesity-related metabolic dysfunction and mitigating MASLD progression [145].

### 3.6. Recommendations for Vitamin D Intake and Considerations for Vulnerable Populations in Metabolic Liver Disease

Vitamin D plays a critical role not only in skeletal health but also in immune regulation, energy metabolism, and hepatic lipid homeostasis [75,97,99]. Adequate vitamin D intake is essential for the general population and becomes particularly relevant in individuals at increased risk of metabolic dysfunction, including obesity, insulin resistance, and MASLD [145]. Given the high prevalence of hypovitaminosis D in metabolically compromised populations, tailored intake recommendations and preventive strategies are necessary to optimize metabolic and hepatic outcomes [145].

Current recommendations for vitamin D intake vary according to age, physiological status, and risk of deficiency [2,164]. For infants, a daily intake of 10 μg is generally advised during the first year of life to support normal skeletal development and immune function. In children and adolescents, vitamin D requirements gradually increase, stabilizing at approximately 15 μg per day from ages 9 to 70 years [2,164]. For adults over 70 years of age, recommended intake increases to 20 μg per day to compensate for reduced cutaneous synthesis and to mitigate age-related risks such as osteoporosis, sarcopenia, frailty, and metabolic decline [2,156]. These age-related recommendations provide a foundational framework but may underestimate the needs of individuals with metabolic disorders [2,164].

Populations at higher metabolic risk, including individuals with obesity, type 2 diabetes, MASLD, or conditions affecting vitamin D absorption and metabolism (e.g., malabsorption syndromes, chronic kidney disease, or liver dysfunction)—often require higher supplementation doses to achieve adequate serum 25(OH)D levels [85,145]. In these groups, daily supplementation ranging from 1000 to 2000 IU is commonly recommended, with dose adjustments based on baseline vitamin D status, body composition, and clinical response. In selected clinical scenarios, higher doses (up to 10,000 IU per day) may be considered under strict medical supervision and with regular biochemical monitoring [2,164]. Attention should be paid to potential drug–nutrient interactions, especially with glucocorticoids, anticonvulsants, and other medications known to interfere with vitamin D metabolism [2,164].

Geographical and environmental factors further modulate vitamin D requirements. Individuals residing in regions with limited UVB exposure, such as higher latitudes, are at increased risk of deficiency, particularly during winter months [2,156,157,158,164]. This risk is compounded in populations with sedentary lifestyles, obesity, or limited outdoor activity—characteristics frequently observed in patients with MASLD [11]. Public health strategies, including food fortification programs and seasonal supplementation, have proven effective in reducing hypovitaminosis D and may indirectly contribute to improved metabolic health at the population level [165].

While vitamin D supplementation offers potential metabolic benefits, safety considerations are essential. The widespread availability of high-dose vitamin D supplements has raised concerns regarding toxicity [166]. Current evidence supports a tolerable upper intake level of 4000 IU per day for most adults [164]. Chronic intake above this threshold, particularly in the absence of biochemical monitoring, increases the risk of hypervitaminosis D, hypercalcemia, and hypercalciuria, which may result in cardiovascular, renal, and neurological complications. High-dose bolus regimens exceeding 300,000 IU have been associated with adverse outcomes and should generally be avoided in routine clinical practice [2,164]. Regular monitoring of serum 25(OH)D and calcium levels is strongly recommended in individuals receiving moderate- to high-dose supplementation, especially those with metabolic or hepatic comorbidities [2,164].

Table 4 summarizes current vitamin D intake recommendations across different population groups, emphasizing tailored strategies for individuals at increased risk due to age, metabolic status, or environmental exposure. Although these recommendations are largely based on guidelines from the Institute of Medicine, alternative positions from the International Osteoporosis Foundation, the American Geriatrics Society, and the Endocrine Society suggest higher intake targets for older adults and individuals with metabolic disorders, including obesity and diabetes. These divergent recommendations highlight the importance of individualized approaches that consider life stage, metabolic health, geographic context, and disease-specific requirements when defining optimal vitamin D intake, particularly in the context of MASLD and related metabolic conditions.

## 4. Limitations of the Current Evidence

Research exploring the relationship between vitamin D and MASLD has expanded considerably in recent years [146]. Nevertheless, important methodological and conceptual limitations continue to hinder a definitive understanding of this association and its translation into clinical practice and public health recommendations [2,164]. Acritical appraisal of these limitations is essential to contextualize current findings and guide future research priorities [19].

One of the main challenges lies in the substantial heterogeneity across study designs. Variability exists in MASLD diagnostic criteria (imaging-based diagnosis versus histological confirmation), disease staging (simple steatosis versus non-alcoholic steatohepatitis), population characteristics, vitamin D dosing regimens, and intervention duration [145]. This heterogeneity complicates data synthesis and limits the ability to draw robust conclusions regarding the causal role of vitamin D in MASLD development or progression [145]. Additionally, many studies rely on cross-sectional designs, which preclude causal inference and fail to capture disease dynamics over time [167].

Vitamin D exerts complex epigenetic effects that may be particularly relevant to hepatic metabolism and fibrogenesis [99]. Through VDR-mediated mechanisms, vitamin D modulates DNA methylation, histone acetylation, and transcriptional regulation of genes involved in insulin signaling, lipid metabolism, and inflammation [18,65,66,72,109,110]. Experimental evidence suggests that vitamin D can influence methylation patterns of genes related to insulin receptor signaling and inflammatory pathways, potentially improving hepatic insulin sensitivity [168]. However, human studies investigating epigenetic modifications in MASLD remain scarce, and the clinical relevance of these mechanisms is not yet fully established [169]. Furthermore, genetic polymorphisms in enzymes involved in vitamin D metabolism, such as *CYP27B1* and *CYP2R1*, may interact with epigenetic regulation, contributing to interindividual variability in response to supplementation [56,57].

Another emerging but insufficiently explored limitation is the role of the gut–liver axis. Vitamin D influences gut microbiota composition by promoting beneficial bacterial taxa while reducing pathogenic species and enhancing intestinal barrier integrity through upregulation of tight junction proteins [170,171,172]. These effects may reduce endotoxemia and hepatic inflammation, processes central to MASLD pathogenesis [170,172]. However, most clinical studies do not assess microbiota-related outcomes, making it difficult to determine whether improvements in hepatic or metabolic parameters are mediated by microbiome modulation or represent indirect effects of vitamin D supplementation [170,172].

At the intracellular level, vitamin D interacts with key metabolic signaling pathways, including AMPK and mTOR, which regulate lipid synthesis, energy sensing, and inflammatory responses in hepatocytes and adipocytes [16,110,111,114,115,159]. Although experimental data suggest that vitamin D may restore AMPK–mTOR balance and attenuate hepatic steatosis, human evidence remains limited and inconsistent [16,107,108,111,112,156,157]. The translation of mechanistic findings into clinically meaningful outcomes is further complicated by differences in study populations, disease severity, and metabolic context [16,110,111,114,115,159].

Methodological inconsistencies in the measurement of serum vitamin D levels represent another major limitation [19]. Assays used to quantify circulating 25-hydroxyvitamin D [25(OH)D], such as immunoassays and high-performance liquid chromatography, vary in accuracy and reproducibility [19]. This lack of standardization undermines comparability across studies and contributes to uncertainty regarding optimal vitamin D thresholds for metabolic and hepatic outcomes [2,19,164]. Moreover, most cutoff values are derived from bone health endpoints and may not reflect the concentrations required to influence hepatic lipid metabolism or inflammation [145].

Confounding variables further obscure interpretation of findings. Factors such as physical activity, dietary intake, body mass index, sun exposure, alcohol consumption, genetic background, and concomitant metabolic disorders strongly influence both vitamin D status and MASLD risk [25,49]. In many studies, these variables are insufficiently controlled, making it difficult to isolate the independent effects of vitamin D [25]. Additionally, obesity-related sequestration of vitamin D in adipose tissue complicates interpretation of serum levels, as low circulating concentrations may reflect altered distribution rather than true deficiency [80,123,125,126,136,139,142].

Most intervention studies focus on short- to medium-term outcomes, leaving uncertainty regarding the long-term effects of vitamin D supplementation on MASLD progression, fibrosis development, or clinical endpoints such as cirrhosis and hepatocellular carcinoma [20,64]. Variability in the form of supplementation (vitamin D_2_ vs. D_3_), dosing strategies, and adherence further limits comparability [173,174,175].

Importantly, the interactive effects of vitamin D supplementation with established lifestyle interventions such as weight loss, dietary modification, physical activity, or pharmacological treatment remain poorly characterized [2,25,164].

From a clinical guideline perspective, no major hepatology or metabolic society currently recommends routine vitamin D supplementation specifically for the prevention or treatment of MASLD, except in cases of confirmed deficiency [145]. Existing recommendations emphasize correction of deficiency rather than targeted metabolic therapy, reflecting the limited and inconsistent evidence base [145]. As a result, vitamin D supplementation remains an adjunctive rather than a primary intervention in MASLD management [145].

Addressing these limitations will require well-designed, long-term randomized controlled trials incorporating standardized vitamin D assessment methods, histologically or imaging-confirmed MASLD phenotyping, and comprehensive evaluation of metabolic, inflammatory, epigenetic, and microbiota-related outcomes [171,172]. Such approaches are essential to clarify the role of vitamin D in MASLD pathogenesis and to determine whether supplementation can provide clinically meaningful benefits beyond correction of deficiency [145].

Table 5 summarizes the current body of evidence examining the association between vitamin D status and MASLD, including observational and interventional studies assessing hepatic steatosis, insulin resistance, inflammatory markers, and disease progression.

## 5. Clinical Implications and Future Research Directions

Despite the growing body of evidence linking vitamin D status to MASLD development and progression, several challenges continue to limit the translation of current findings into clinical practice. A major obstacle is the substantial interindividual genetic variability affecting vitamin D signaling, particularly polymorphisms in the vitamin D receptor (VDR) and enzymes involved in its activation and degradation (e.g., *CYP27B1* and *CYP24A1*). These genetic differences influence hepatic, adipose, and immune responses to vitamin D, complicating the extrapolation of results across heterogeneous populations and contributing to inconsistent clinical outcomes.

Methodological limitations further constrain clinical applicability. Considerable variability persists in the assessment of circulating 25-hydroxyvitamin D [25(OH)D], with discrepancies between immunoassays and mass spectrometry-based techniques undermining cross-study comparability. In addition, the lack of standardized thresholds for vitamin D deficiency and sufficiency in the context of liver disease complicates patient stratification and therapeutic decision-making. Most available studies are cross-sectional or short-term interventions, limiting insight into the long-term effects of vitamin D on hepatic steatosis resolution, fibrosis progression, and transition from simple steatosis to MASH.

Another critical limitation is the incomplete control of confounding factors that strongly influence both vitamin D status and MASLD severity, including adiposity, insulin resistance, dietary patterns, physical activity, sun exposure, and concurrent pharmacological treatments. These factors obscure causal inference and highlight the need for more rigorous study designs. Future research should prioritize well-controlled, longitudinal trials that integrate comprehensive metabolic phenotyping and account for lifestyle and environmental determinants.

From a clinical perspective, advancing toward personalized medicine is essential. Stratification based on genetic background, sex, ethnicity, and metabolic phenotype may help identify subgroups most likely to benefit from vitamin D supplementation. Standardization of study protocols—including dosage, formulation (vitamin D_2_ vs. D_3_), duration, and co-interventions—is equally critical. Emerging technologies offer promising avenues to deepen mechanistic understanding: CRISPR-Cas9–based models can elucidate the functional role of VDR signaling in hepatocytes and adipocytes, while proteomics and metabolomics may identify sensitive biomarkers reflecting hepatic lipid handling, inflammation, and fibrogenesis.

Future trials should also explore synergistic strategies, assessing vitamin D supplementation in combination with lifestyle interventions such as structured exercise or dietary modification, given their shared effects on insulin sensitivity, adipose tissue function, and hepatic lipid flux. In populations at higher risk of deficiency—such as individuals with obesity, limited sunlight exposure, or advanced liver disease—tailored and culturally appropriate supplementation strategies are warranted. Additionally, evaluating interactions between vitamin D and other micronutrients (e.g., calcium or magnesium) may further optimize metabolic and hepatic outcomes.

Collectively, addressing these methodological and biological challenges will be crucial for defining the true clinical role of vitamin D in MASLD prevention and management. Robust, standardized, and mechanistically informed research will ultimately determine whether vitamin D can be integrated as an adjunctive strategy within evidence-based approaches to mitigate hepatic steatosis, inflammation, and disease progression.

## 6. Conclusions

Vitamin D signaling emerges as a multifaceted regulator of key molecular pathways involved in MASLD pathogenesis, including insulin resistance, lipid metabolism, oxidative stress, inflammation, and fibrogenesis. While experimental evidence strongly supports a protective role for vitamin D–VDR activation in the liver, clinical outcomes remain heterogeneous. Future well-designed trials integrating disease stage, baseline vitamin D status, and molecular biomarkers are required to clarify its therapeutic potential. Understanding the role of vitamin D within the liver–adipose–immune axis may open new avenues for targeted interventions in MASLD.

## Figures and Tables

**Table 1:** Diagnostic criteria and risk factors for MASLD.

References	Category	Factor	Criteria	Diagnostic Instruments
[49]	Diagnostic Criteria ^1^	Hepatic steatosis	≥5% steatosis by imaging or histology	Ultrasound and liver biopsy
[49]	Mandatory Exclusion ^1^	Alcohol consumption	<20 g/day (women) <30 g/day (men)	Structured Questionnaire
		Other liver diseases	Viral, autoimmune, genetic, drug-induced excluded	Viral serology, autoimmune markers, iron studies
[49]	Disease severity ^1^	MASLD (histological)	Steatosis + inflammation + hepatocyte ballooning ± fibrosis	Liver biopsy (NAS or SAF)
[50]	Fibrosis staging ^1^	Significant fibrosis	≥F2 histology or non-invasive assessment	Biopsy (METAVIR), transient elastography, FIB-4
	Metabolic risk factors ^1,2^	Overweight/Obesity	BMI ≥ 25 kg/m ^2^ (ethnicity-adjusted) or increased waist circumference	Measured BMI and waist circumference (WHO/IDF cut-offs)
[31,49,50,51]		Type 2 diabetes mellitus	Established diagnosis or HbA1c ≥ 6.5%	HbA1c, fasting glucose
		Metabolic syndrome	≥3 standard metabolic syndrome criteria	NCEP-ATP III or IDF criteria
		Dyslipidemia	Elevated triglycerides and/or low HDL	Fasting lipid profile
		Hypertension	≥130/85 mmHg or treatment	Standardized BP measurement

^1^ Based on recommendations from AASLD, EASL, and APASL clinical practice guidelines. ^2^ MASLD diagnosis requires steatosis plus exclusion of secondary causes; presence of metabolic risk factors strongly supports diagnosis but is not mandatory in all cases.

**Table 2:** Key molecular regulators involved in MASLD pathogenesis.

References	Gene/Protein	Function	Characteristics
[147]	SREBP-1c	Master regulator of de novo lipogenesis	Induces ACC and FASN expression; promotes hepatic triglyceride synthesis
[148]	PPARα	Regulates fatty acid β-oxidation	Enhances mitochondrial and peroxisomal lipid oxidation; protective against steatosis
[147,148]	PPARγ	Modulates lipid storage and insulin sensitivity	Upregulated in steatotic liver; promotes lipid uptake and storage
[78]	ChREBP	Glucose-responsive lipogenic transcription factor	Activated by high glucose; stimulates glycolysis and lipogenesis
[147]	AMPK	Energy sensor regulating lipid metabolism	Inhibits lipogenesis; activates fatty acid oxidation; suppresses gluconeogenesis
[149]	mTORC1	Nutrient-sensing regulator of anabolic pathways	Promotes lipogenesis and inhibits autophagy when overactivated
[150]	NF-κB	Central mediator of inflammation	Induces TNF-α, IL-6; contributes to insulin resistance and MASH progression
[151]	TGF-β	Key profibrogenic cytokine	Activates hepatic stellate cells; drives extracellular matrix deposition
[152]	CYP2E1	Oxidative stress mediator	Generates reactive oxygen species; contributes to lipid peroxidation
[153]	Adiponectin (ADIPOQ)	Insulin-sensitizing adipokine	Activates AMPK; reduced levels associated with steatosis and inflammation

**Table 3:** Interpretation of serum 25-hydroxyvitamin D [25(OH)D] levels according to selected international organizations.

Vitamin D Status	Netherlands (Health Council)	Institute of Medicine (IOM/NAM)	IOF & American Geriatrics Society	Expert Opinion
Severe deficiency	10–12 ng/mL 25–30 nmol/L			
Deficiency	NR	<20 ng/mL <50 nmol/L	<30 ng/mL <75 nmol/L	<40 ng/mL <100 nmol/L
Sufficiency	>10–12 ng/mL >25–30 nmol/L	>20 ng/mL >50 nmol/L	>30 ng/mL >75 nmol/L	>40 ng/mL >100 nmol/L

NR. No report. Source: adaptation of Herrera-Molina et al. [19].

**Table 4:** Recommended dietary vitamin D intake according to the Institute of Medicine (IOM/NAM) and the Endocrine Society.

Age Group (Years)	IOM—AI (μg/IU)	IOM—EAR (μg/IU)	IOM—RDA (μg/IU)	IOM—UL (μg/IU)	Endocrine Society Recommended (IU/Day)	Endocrine Society—UL (IU/Day)
0–6 months	10/400	-	-	25/1000	400–1000	2000
6–12 months	10/400	-	-	38/1500	400–1000	2000
1–3	-	10/400	15/600	63/2500	600–1000	4000
4–8	-	10/400	15/600	75/3000	600–1000	4000
9–13	-	10/400	15/600	100/4000	600–1000	4000
14–18	-	10/400	15/600	100/4000	600–1000	4000
19–30	-	10/400	15/600	100/4000	1500–2000	10,000
31–50	-	10/400	15/600	100/4000	1500–2000	10,000
51–70	-	10/400	15/600	100/4000	1500–2000	10,000
>70	-	10/400	20/800	100/4000	1500–2000	10,000

AI: Adequate Intake, EAR: Estimated Average Requirement, RDA: Recommended Dietary Allowances, UL: Tolerable upper intake level, IU: International Units, μg: microgram. Source: adaptation of Demay et al. [2].

**Table 5:** Summary of current evidence on MASLD.

References	Purpose	Hazard Ratio (95% CI)
Jaruvongvanich et al. [145]	Association between serum vitamin D levels and MASLD histologic severity (NAS and fibrosis score)	High NAS vs. Low NAS: MD = −0.93 (95% CI: −2.45 to 0.58); I^2^ = 0%
		High fibrosis vs. Low fibrosis: MD = 0.88 (95% CI: −2.65 to 4.42); I^2^ = 64%
Guo et al. [176]	Effect of supplemental vitamin D on metabolic and liver markers in MASLD patients.	Fasting glucose: SMD = −0.22 (95% CI: −0.39 to −0.04); I^2^ = 4.3%
		Insulin: SMD = −0.68 (95% CI: −1.22 to −0.14); I^2^ = 85.2%
		HOMA-IR: SMD = −1.32 (95% CI: −2.30 to −0.34); I^2^ = 94.8%
		ALT: SMD = −0.18 (95% CI: −0.39 to 0.04); I^2^ = 38.8%
		TAG: SMD = −10.38 (95% CI: −21.09 to 0.34); I^2^ = 37.5%
Bjelakovic et al. [177]	Effects of vitamin D supplementation on adults with chronic liver diseases	All-cause mortality: OR = 0.70 (95% CI: 0.09 to 5.38); I^2^ = 32%
		Liver-related mortality: RR = 1.62 (95% CI: 0.08 to 34.66); I^2^ = N/A.
		Hypercalcaemia: RR = 5 (95% CI: 0.25 to 100.8); I^2^ = N/A
		Myocardial infarction: RR = 0.75 (95% CI: 0.08 to 6.81); I^2^ = 0%
		Thyroiditis: RR = 0.33 (95% CI: 0.01 to 7.91); I^2^ = N/A
		Glossitis: RR = 3.70 (95% CI: 0.16 to 87.6)
Liu et al. [178]	Association between serum vitamin D levels and risk of MASLD	MASLD: SMD = −0.90 (95% CI: −1.29 to −0.52); I^2^ = 99%
		MASLD risk: OR = 0.64 (95% CI: 0.54 to 0.77); I^2^ = 99%
		Western populations: OR = 0.60 (95% CI: 0.46 to 0.78); I^2^ = 99%
Eliades et al. [179]	Association between serum vitamin D levels and MASLD	MASLD low levels of 25(OH)D (0.36 ng/mL): SMD = −0.36 (95% CI: −0.32 to −0.40); I^2^ = 99.2%
		MASLD and likelihood of vitamin D deficiency: OR = 1.26 (95% CI: 1.17 to 1.35); I^2^ = 65.2%
Zhu et al. [180]	Association between serum vitamin D levels and MASLD in children and adolescents	MASLD had lower 25(OH)D levels: SMD = −0.59 (95% CI: −0.98 to −0.20); I^2^ = 89.8%
Sindhughosa et al. [181]	Effect of vitamin D supplementation on insulin resistance in MASLD patients	HOMA-IR: MD = −1.06 (95% CI: −1.66 to −0.45); I^2^ = 67%
		Serum 25(OH)D: MD = 17.45 (95% CI: 8.33 to 26.56); I^2^ = 98%
		ALT: MD = −4.44 (95% CI: −8.24 to −0.65); I^2^ = 51%
		AST: MD = −5.28 (95% CI: −12.34 to 1.79); I^2^ = 83%
Mansour-Ghanaei et al. [182]	Effect of vitamin D supplementation on liver enzymes in MASLD patients	ALT: MD = −2.88 (95% CI: −6.03 to 0.27); I^2^ = 85%
		AST: MD = −0.10 (95% CI: −1.18 to 0.97); I^2^ = 26%
		γ-GT: MD = 0.12 (95% CI: −5.94 to 6.18); I^2^ = 38%
		ALP: MD = −13.79 (95% CI: −22.13 to −5.45); I^2^ = 72%
		Subgroup (>3000 IU/day) ALP: MD = −19.74 (95% CI: −25.36 to −14.12); I^2^ = 0%
Chen et al. [183]	Effect of vitamin D supplementation on metabolic parameters in MASLD patients	25(OH)D: MD = 2.01 (95% CI: 0.94 to 3.08); I^2^ = 96%
		HOMA-IR: MD = −0.54 (95% CI: −1.28 to 0.20); I^2^ = 92%
		Fasting glucose: MD = −0.59 (95% CI: −1.50 to 0.32); I^2^ = 94%
		FINS: MD = −0.30 (95% CI: −0.77 to 0.17); I^2^ = 81%
Tabrizi et al. [184]	Effect of vitamin D supplementation on metabolic profiles and liver function in MASLD patients	FPG: SMD = −0.23 (95% CI: −0.88 to 0.42); I^2^ = 88,2%
		Insulin: SMD = −1.09 (95% CI: −2.70 to 0.52); I^2^ = 96.9%
		HOMA-IR: SMD = −1.89 (95% CI: −3.88 to 0.09); I^2^ = 97.6%
		Triglycerides: SMD = −0.36 (95% CI: −1.77 to 1.04); I^2^ = 96.5%
		Total cholesterol: SMD = −0.46 (95% CI: −1.3 to 0.39); I^2^ = 91.6%
		ALT: SMD = −0.66 (95% CI: −1.43 to 0.11); I^2^ = 88.8%
		AST: SMD = −0.53 (95% CI: −1.11 to 0.05); I^2^ = 80.9%
		BMI: SMD = −0.25 (95% CI: −0.76 to 0.27); I^2^ = 81.8%
Yuan et al. [185]	Investigate causal association between serum 25(OH)D and MASLD using Mendelian randomization	MASLD predisposition associated vitamin D ST = 0.78 (95% CI: 0.69 to 0.89)
		AST: ST = −1.17 (95% CI: −1.36 to 0.01)
		MASLD predisposition not associated vitamin D: ST = 0.13 (95% CI: −1.26 to 0.53)

MASLD = Metabolic dysfunction-associated steatotic liver disease; NAS = NAFLD Activity Score; ALT = Alanine aminotransferase; AST = Aspartate aminotransferase; ALP = Alkaline phosphatase; γ-GT = Gamma-glutamyl transferase; FPG = Fasting plasma glucose; FINS = Fasting insulin; HOMA-IR = Homeostatic Model Assessment of Insulin Resistance; SMD = Standardized mean difference; MD = Mean difference; OR = Odds ratio; RR = Risk ratio; I^2^ = Heterogeneity statistic; 25(OH)D = 25-hydroxyvitamin D; TAG = Triglycerides; BMI = Body mass index; ST = Standardized effect (used in Mendelian randomization studies).

## Data Availability

The data related to this study are available in this article.
